# Part 3. Triethylborane-air: a suitable initiator for intermolecular radical additions of *S*-2-oxoalkyl-thionocarbonates (*S*-xanthates) to olefins

**DOI:** 10.1186/1860-5397-3-47

**Published:** 2007-12-13

**Authors:** Jean Boivin, Van Tai Nguyen

**Affiliations:** 1 Institut de Chimie des Substances Naturelles, C.N.R.S., Avenue de la Terrasse, 91198 Gif-sur-Yvette, France; 2 National Institute of Medicine Materials, 3B, Quang Trung, Hanoi, Vietnam

## Abstract

Under carefully controlled conditions, the triethylborane-air combination proves to be an efficient radical initiator that allows intermolecular radical additions of *S*-2-oxoalkyl-thionocarbonates (*S*-xanthates) to olefins. Depending on both the structures of the xanthate and the olefin, the addition process can be achieved at room temperature or slightly higher.

## Background

Alkylboranes, mainly triethylborane, have become more and more popular as radical initiators because of their ability to generate alkyl radicals by reaction with dioxygen (or air) even at very low temperature (–78°C). [1–4] To the best of our knowledge, only one attempt to use Et_3_B as a radical initiator at 0°C for the intermolecular addition of an *S*-alkylxanthate onto 1,1-dimethoxy-2-cyclopropene, has been mentioned in the literature but without success.[5–6] In part 1 of this series, we reported that trialkylboranes are convenient reagents, when used in excess, to reduce *S*-alkyl-thionocarbonates (*S*-xanthates), *O*-alkyl-thionocarbonates (*O*-xanthates) and related compounds to the corresponding alkanes at room temperature.[7] In the present article, we wish to report that a more comprehensive understanding of the different routes involved permits the premature reduction of the starting 2-oxoalkylxanthate to be avoided. Then, by carefully choosing the *modus operandi*, the transient α-acyl carbon radical can then be trapped by a suitable olefin, thus offering a mild and efficient method to achieve intermolecular radical additions. In a recent paper, Zard described additions of various *S*-alkylxanthates to vinyl epoxides and related derivatives using an excess of triethylborane (2 equiv *vs* xanthate) at room temperature. The mechanism is different from that reported in this note as the radical chain is maintained by the ring opening of the oxirane that produces an alkoxy radical. The latter reacts rapidly with Et_3_B to afford a borinate and ethyl radical.[8]

## Results and discussion

**Scheme 1:**
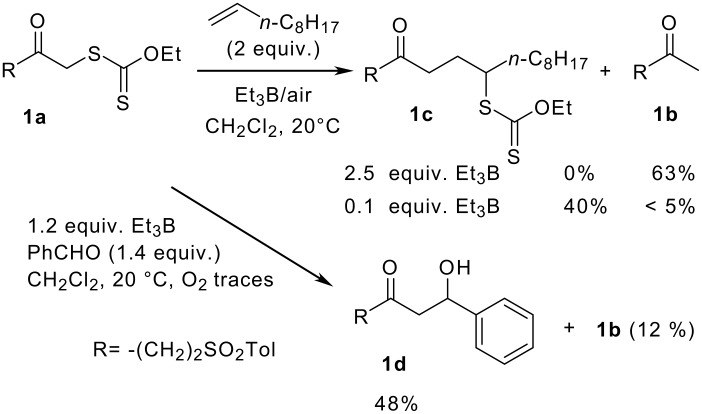
Reactivity of 2-oxoalkylxanthates toward 1-decene in the presence of Et_3_B/O_2_: competition between addition and reduction.

The pivotal experiments at the origin of this paper are depicted in Scheme 1. In the first experiment, 2.5 equiv of Et_3_B were added to a mixture of xanthate **1a** and 1-decene in dichloromethane under argon at 20°C. The stopper was then removed and air was allowed to enter the flask. After 1 h, purification afforded reduced starting material **1b** as the only isolated compound (63%). Such a reactivity was not surprising in view of previous observations [3] and from the literature data. [9–14] Trapping with benzaldehyde gave aldol **1d** (48%, Scheme 1) and thus confirmed that a boron enolate is a plausible intermediate in the reduction of compound **1a** into **1b**. One cannot put aside the possibility that the reduction of the transient α-acyl radical may also occur, to a minor extent, *via* a direct transfer from a hydrogen donor.[4,15]

Scheme 1 shows that xanthate **1a** undergoes two main types of reactions. The group transfer reaction operates through a radical chain mechanism and affords the adduct **1c**.[16] The reduction of compound **1a** into **1b** results from a bimolecular process in which Et_3_B is implicated not only in the generation of the α-acyl radical but also in the reaction with the latter, in a stoichiometric manner, to afford an intermediate boron enolate. Lowering the amount of Et_3_B would therefore minimise the premature unwanted reduction. This hypothesis was then tested. When Et_3_B (0.1 equiv) was used in catalytic amounts, adduct **1c** was isolated in a modest but remarkable 40% yield (Scheme 1), together with traces of compound **1b** (<5%). We reasoned that a slow addition of Et_3_B would diminish more efficiently the unwanted reduction into **1b**. On the other hand, slow addition of air would maintain a low concentration of the radical species and hence minimise the usual unwanted side reactions (dimerisations, abstractions...) that could hamper a clean addition process. Accordingly, as Et_3_B was added slowly with a syringe pump to a 0.4 M solution of xanthate **1a** (0.6 mmol) and 1-decene (2 equiv) in dichloromethane at 20°C, air was injected (10 mL/h) at the same time in the reaction medium with a second syringe pump [see Supporting Information File 1]. The data reported in Table 1 show that, with this technique, diminishing the rate of addition of Et_3_B from 0.15 mmol/h to 0.03 mmol/h and increasing simultaneously the total amounts of Et_3_B (from 0.2 to 0.4 equiv) resulted in a marked improvement. The yield of adduct **1c** increased from 35 to 64%. At the same time, the amount of recovered **1a** dropped from 30 to 11% and the reduction into compound **1b** was totally suppressed.

**Table 1:** Addition of xanthate 1a to decene at r.t., catalysed by Et_3_B/air

entry	Et_3_B mmol/h (equiv)	Time (h)	Decene (equiv)	**1c** (%)^a^	**1b** (%)

1	0.15 (0.2)	1.1	2	35 (30)	11
2	0.06 (0.3)	3.15	2.5	47 (21)	6
3	0.03 (0.4)	7.3	2.5	64 (11)	-

^a^ in parentheses, percentage of recovered starting material **1a**

**Figure 1:**
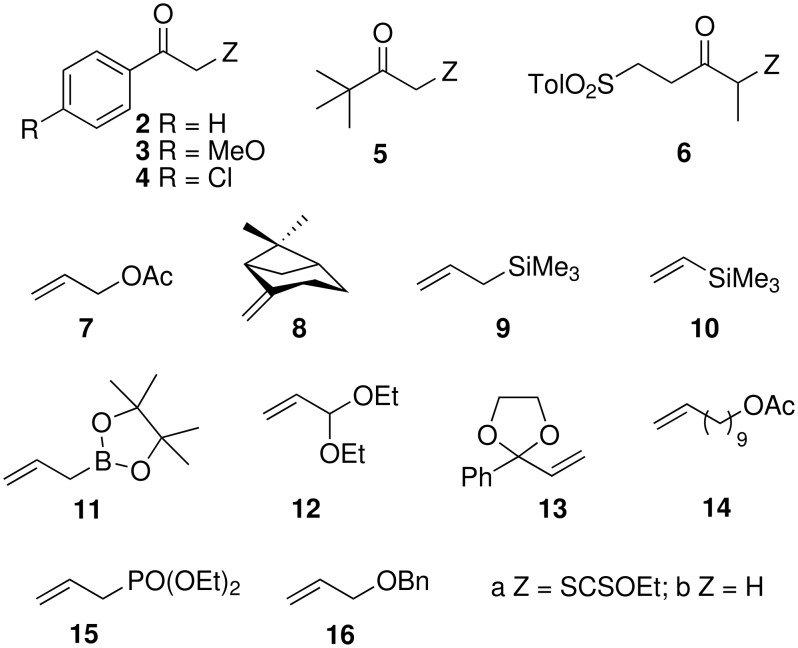
Starting xanthates and olefins.

**Figure 2:**
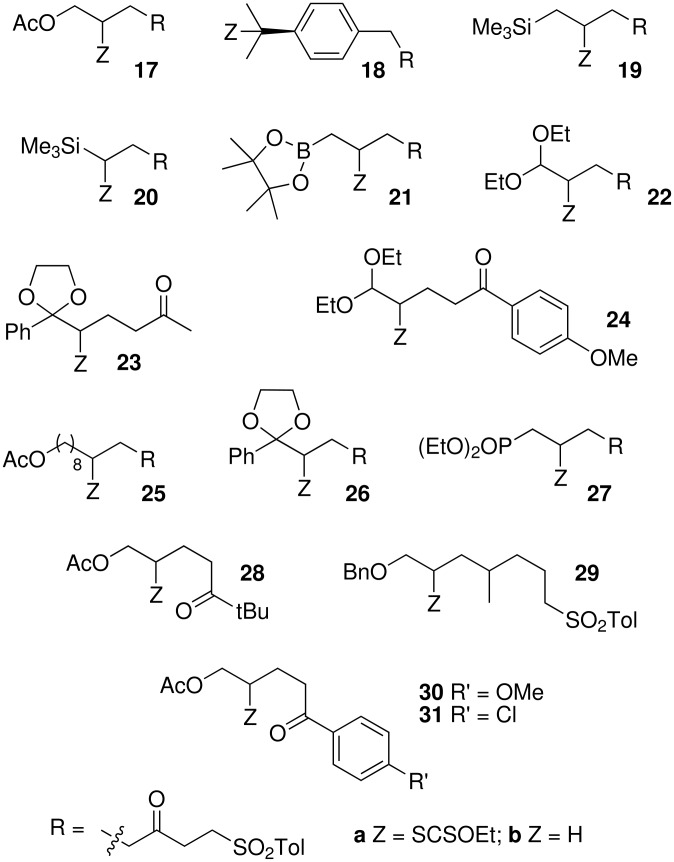
Adducts between xanthates and olefins.

Using this procedure, xanthate **1a** was added to various olefins (Figure 1 and Figure 2, Table 2). Addition to allyl acetate (**7**) furnished adduct **17a** in 51% yield accompanied by some starting material **1a** (entry 1) [see Supporting Information File 2]. Addition to pinene (**8**) gave compound **18a** (44%) and some reduced adduct **18b** (12%, entry 2). Interestingly, addition of xanthate **1a** to allylsilane **9** gave adduct **19a** in a high yield (71%, entry 3), while addition of xanthate **1a** to vinylsilane **10** afforded adduct **20a** (57%, entry 4). Reaction of xanthate **1a** with allylboronate **11** gave compound **21a** in a modest yield (41%, entry 5). Similarly, addition to acrolein diethyl acetal (**12**), at 20°C, led to the desired compound **22a** in a low yield (25%), and much starting material **1a** (60%) was recovered. A small amount of reduced compound **1b** was also isolated (5%, entry 6).

**Table 2:** Et_3_B/air catalysed intermolecular radical additions to olefins

Entry	Xanthate	Olefin (equiv)	Et_3_B (equiv)	Solvent	T (°C)	Time (h)	Products (yields %)

1	**1a**	**7** (2.5)	0.5	CH_2_Cl_2_	20	8	**17a** (51); **1a** (20)
2	**1a**	**8** (2.5)	0.5	CH_2_Cl_2_	20	8	**18a** (44); **18b** (12)
3	**1a**	**9** (2.5)	0.5	CH_2_Cl_2_	20	8	**19a** (71)
4	**1a**	**10** (2.5)	0.5	CH_2_Cl_2_	20	8	**20a** (57)
5	**1a**	**11** (2.5)	0.5	CH_2_Cl_2_	20	8	**21a** (41)
6	**1a**	**12** (2.5)	0.5	CH_2_Cl_2_	20	8	**22a** (25); **1b** (5); **1a** (60)
7	**2a**	**13** (2.5)	0.5	CH_2_Cl_2_	20	8	**23a** (0); **2a** (41)
8	**3a**	**12** (2.5)	0.5	CH_2_Cl_2_	20	8	**24a** (0); **3a** (54); **3b** (12)
9	**2a**	**13** (2)	0.3	CH_2_Cl_2_	40	8	**23a** (51); **2b** (12)
10	**3a**	**12** (3)	0.5	CH_2_Cl_2_	40	8	**24a** (47); **3b** (26)
11	**3a**	**7** (2.5)	0.5	CH_2_Cl_2_	40	8	**30a** (49); **3b** (39)
12	**4a**	**7** (5)	0.4	CH_2_Cl_2_	40	7	**31a** (45); **4b** (34)
13	**1a**	**12** (2.5)	0.3	CH_2_Cl_2_	40	6	**22a** (74)
14	**1a**	**14** (2)	0.3	CH_2_Cl_2_	40	8	**25a** (52); **25b** (20)
15	**1a**	**13** (2)	0.3	CH_2_Cl_2_	40	8	**26a** (66)
16	**1a**	**9** (2.5)	0.3	CH_2_Cl_2_	40	4	**19a** (77) (71)^c^
17	**1a**	**10** (2.5)	0.3	CH_2_Cl_2_	40	4	**20a** (42)
18	**1a**	**11** (2.5)	0.3	CH_2_Cl_2_	40	4	**21a** (54)
19	**1a**	**15** (2.5)	0.3	CH_2_Cl_2_	40	4	**27a** (59)
20	**1a**	**10** (2.5x2)	0.3	CH_2_Cl_2_	40	4	**20a** (73)^d^
21	**5a**	**7** (2)	0.1^b^	(CH_2_Cl)_2_	83	4	**28a** (77)^a^
22	**6a**	**16** (2)	0.4^b^	(CH_2_Cl)_2_	83	14	**29a** (54)
23	**1a**	**7** (2)	0.1^b^	(CH_2_Cl)_2_	83	4	**17a** (78)
24	**1a**	**8** (2)	0.15^b^	(CH_2_Cl)_2_	83	6	**18a** (79)
25	**3a**	**7** (2)	0.1^b^	(CH_2_Cl)_2_	83	4	**30a** (71)
26	**4a**	**7** (2)	0.1^b^	(CH_2_Cl)_2_	83	4	**31a** (73)
27	**3a**	**12** (2)	0.15^b^	(CH_2_Cl)_2_	83	4	**24a** (60)

^a^ When the reaction was performed at 40°C, no adduct was formed. The starting material **7a** was recovered. ^b^ Et_3_B (1 M solution in hexanes) was added with a syringe pump (0.03 mmol/h). ^c^ reaction flask equipped with a condenser opened to air. ^d^ The second portion of vinyltrimethylsilane (2.5 equiv) was added after 90 min.

We then turned our attention to the highly delocalised radicals derived from aromatic ketones **2a**, **3a**, and **4a**. As anticipated, this represented one of the worst situations, as premature reduction to methyl ketone should be relatively fast when compared to intermolecular addition to an olefin. Experiments 7 and 8 validated this hypothesis. When xanthate **2a** was reacted at 20°C with phenylvinyl dioxolane **13** in the presence of Et_3_B, no trace of the adduct **23a** could be isolated, and only starting material **2a** was recovered (Table 2, entry 7). Under the same conditions, xanthate **3a** also failed to add to acrolein diethyl acetal (**12**) (entry 8). The only compounds that could be isolated were the starting material **3a** (54%) and the reduced product **3b** (12%). Obviously, the reaction conditions needed to be adjusted in order to favour the addition process with regard to both premature reduction of the starting material and useless degenerate reaction of the α-acyl carbon radical with its precursor.[8]

We were delighted to observe that gently warming the reaction in refluxing dichloromethane (40°C) totally turned the course of the reaction. Thus, xanthate **2a** added to olefin **13** in a fair 51% yield (entry 9). All the starting material was consumed and only 12% of acetophenone (**2b**) were formed. Similarly, xanthate **3a**, in the presence of olefin **12**, succeeded in giving adduct **24a** (47%, entry 10) accompanied by some *p*-methoxyacetophenone (**3b**) (26%). For these two substrates, comparison between experiments 7–10 showed a striking effect of the temperature on the outcome of the reaction: at 20°C no addition was observed but simply warming the reaction medium to 40°C ensured a clean intermolecular addition process. Under the same conditions, α-phenacyl xanthates **3a** and **4a** were also reacted with allyl acetate (entries 11 and 12). In both cases the corresponding adducts were the major products (**30a** and **31a**, 49% and 45% yield, respectively), accompanied by some reduced starting materials (39% and 34% respectively). The astonishing effect of the temperature increase from 20 to 40°C noticed with the aromatic ketones also held for "normal" ketones, albeit to a less dramatic extent. Thus, xanthate **1a** condensed with olefin **12** with a much higher yield (74%, entry 13) than at 20°C (25%, entry 6). Xanthate **1a** also reacted with olefins **14** (entry 14) and **13** (entry 15) to afford adducts **25a** and **26a** in satisfactory 52% and 66% yields, respectively. Similarly, **1a** condensed with olefins **9, 10, 11** and **15** to furnish adducts **19a, 20a, 21a**, and **27a** in 77%, 42%, 54% and 59% yield respectively (entries 16–19). In the case of addition to vinyltrimethylsilane (**10**), the yield obtained at 40°C, lower than the one observed at 20°C, is clearly due to the volatility of vinyltrimethylsilane, as demonstrated by experiment 20 where the addition of more olefin (2.5 equiv) during the reaction resulted in a marked increase of the yield (73%). It is important to note that, at 40°C, no trace of prematurely reduced starting material **1b** could be isolated.

Nevertheless, some substrates were still refractory. Thus, at 40°C in dichloromethane, xanthate **5a** failed to add to allyl acetate and was recovered unchanged. However, when the reaction was performed in refluxing 1,2-dichloroethane (83°C), adduct **28a** was isolated in an excellent 77% yield (Table 2, entry 21). Under the same conditions, secondary xanthate **6a** reacted cleanly with allyl benzyl ether (**16**) to give compound **29a** in 54% yield (Table 2, entry 22). We re-examined reactions that gave moderate yields at 20 or 40°C. In all cases, the yields were markedly improved (compare entries 1 *vs* 23, and entries 2 *vs* 24) even for less reactive aromatic ketones (compare entries 8 *vs* 10, entries 11 *vs* 25, and entries 12 *vs* 26).

When the addition was carried out at 83°C, the reaction time was shorter and the amounts of Et_3_B could be lowered to only 0.10–0.15 equiv *vs* the starting xanthate (entries 21, 23, 25, 26, and 27).

**Scheme 2:**
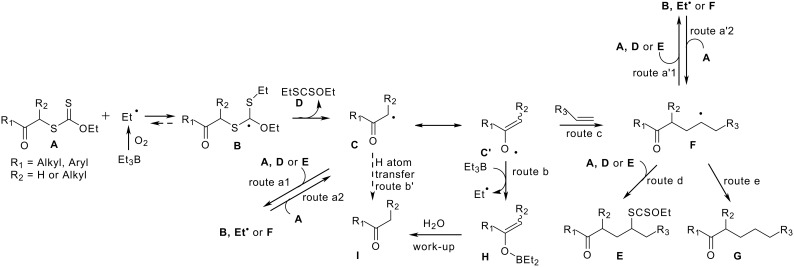
Postulated mechanism for the reaction of 2-oxoalkyl xanthates with olefins in the presence of Et_3_B.

From a mechanistic viewpoint, the results reported herein may be rationalised as follows (Scheme 2). The initiation of the process is governed by interaction of dioxygen with Et_3_B to give Et•. This reaction occurs within a wide range of temperatures. The reaction of ethyl radical with the highly radicophilic species **A** leads to stabilised radical **B**. The latter fragments either to xanthate **A** and Et• or, more easily, to stabilised α-acyl radical **C** and dithiocarbonate **D**. From the intermediate radical **C**, three possible routes determine the outcome of the reaction. Route a_1_ represents the xanthate group transfer between radical **C** and any *O*-ethyl dithiocarbonate (**A**, **D**, or **E**) present in the reaction mixture. The xanthate group transfer (route a_2_) leads to the formation of **C**. Routes a_1_, a_2_ (and routes a'_1_ and a'_2_, see below) constitute a body of fast but useless processes (degenerate reactions)[16] that preserve the radical character but do not let the system evolve. Route c is a relatively slow reaction when compared to the degenerate reactions or to reaction of Et• with **A**. Routes b and b' depend on the concentration of Et_3_B that can be controlled by maintaining a low concentration of Et_3_B. The addition to olefins (route c) is practically irreversible because of the formation of a strong C-C bond. However, the efficiency of route c, compared to routes b, b' and a_1_, is strongly linked to the structures of both xanthate **A** and olefin R_3_-CH=CH_2_, and can be dramatically modified by varying the reaction temperature. Fortunately, such an increase of reaction temperature enhances route c much more than route b.

For planar radicals, Rüchardt and Beckwith established that the C-H bond dissociation energy (BDE) for H-CXYZ compounds displays a good linear correlation with the measured α and β-proton ESR hyperfine splitting constants. [17–19] When Y = Me, Z = H, the H-C BDE follows the order for X: CH=CH_2_ < Ph < PhCO = MeCO < CN < CO_2_Et < Me (Table 3, entries 1–7). The BDE for compounds where Y = Z = H follows the same order, albeit the value is of course slightly higher when compared to their methylated counterparts (entries 8–11). On the other hand, trialkylboranes react much faster with an oxygen centered radical [20–22] than with a carbon radical.[23–24] Therefore, when R_1_ = aryl, the highly stabilised and delocalised radical **C** <--> **C'** (R_2_CH•-COAr <--> R_2_CH=CO•Ar) has a strong propensity to react with Et_3_B on the oxygen part where a high electron density is located, thus affording the enolboronate **H** (Scheme 2). Therefore it is not surprising that the rare literature reports of successful intermolecular radical additions of α-oxo carbon radicals are limited to esters[2–3] that correspond to less stabilised radicals more likely to react on the carbon centre.

**Table 3:** Selected values of H-C BDE for compounds H-CXYZ from references [17–19].

Entry	X	Y	Z	C-H BDE (kcal mol^-1^)

1	Me	Me	H	95.7
2	CO_2_Et	Me	H	95.6
3	CN	Me	H	94.9
4	PhCO	Me	H	92.9 (91)^a^
5	EtCO	Me	H	91.2^a^
6	Ph	Me	H	90.3
7	CH=CH_2_	Me	H	86.1
8	COMe	H	H	97
9	PhCO	H	H	96^a^
10	Ph	H	H	91^a^
11	CH=CH_2_	H	H	88.8^a^

^a^ Determined according to Bordwell's method.

Nevertheless, we have shown in this article that an efficient control of the various reaction parameters (slow additions, temperature) permitted us to elude this problem. For α-oxo carbon radicals derived from aliphatic ketone derivatives, we succeeded in reducing this impediment, and the usual intermolecular addition could take place readily, even at low temperature.

In a previous paper, [7] we showed that the Et_3_B/air combination efficiently promotes the reduction of *S*-alkylxanthates. It is an apparent paradox that, even when "large" amounts of Et_3_B were used (i.e. 0.3–0.5 equiv, entries 1–20 and 22), the reduced product **G** could be detected only in a few instances (entries 2 and 14). However, in the addition process described in this paper, contrary to the reduction method (see ref. [7]), the concentration of Et_3_B is maintained very low by slow addition with a syringe pump, thus minimising route e. Moreover, we demonstrated that the reduction process is relatively slow. As a consequence, the "degenerate" route a'_1_ is much more efficient than the route e.

## Conclusion

We have described a new, efficient, and extremely mild method for performing radical additions of 2-oxoalkylxanthates to various olefins. The efficiency of the addition process *vs* the premature reduction depends on the reactivity of a particular substrate toward a specific olefin for given reaction conditions. This approach can be extended to cyclisations that should operate even at low temperature.

## Supporting Information

File 1 General procedure for intermolecular radical additions of *S*-2-oxoalkyl-thionocarbonates to olefins, reaction at room temperature.

File 2 Part 1. Reduction of *S*-alkyl-thionocarbonates and related compounds in the presence of trialkylboranes/air. Detailed procedures for preparation of new compounds and their spectroscopic data.
