# Supplementary material for: Part 3. Triethylborane-air: a suitable initiator for intermolecular radical additions of S-2-oxoalkyl-thionocarbonates (S-xanthates) to olefins
Source: Beilstein J Org Chem. 2007 Dec 13;3:47. doi: 10.1186/1860-5397-3-47 (PMC2213663; doi:10.1186/1860-5397-3-47)
Supplement: File 1 — General procedure for intermolecular radical additions of S-2-oxoalkyl-thionocarbonates to olefins, reaction at room temperature. [file Beilstein_J_Org_Chem-03-47-s001.pdf]

Part 3.Triethylborane-air: a suitable initiator for intermolecular radical additions of *S*-2-oxoalkyl-thionocarbonates (*S*-xanthates) to olefins

Jean Boivin*[1] and Van Tai Nguyen*[1,2]

Addresses: [1]Institut de Chimie des Substances Naturelles, C.N.R.S., Avenue de la Terrasse, 91198 Gif-sur-Yvette, France, [2]Present address: National Institute of Medicine Materials, 3B, Quang Trung, Hanoi, Vietnam

Email: Jean Boivin*- jean.boivin@icsn.cnrs-gif.fr; Van Tai Nguyen* - htqt.imm@hn.vnn.vn

General procedure for intermolecular radical additions of S-2-oxoalkyl-thionocarbonates to olefins, reaction at room temperature

Triethylborane (solution 1M in hexanes) was injected with the aid of a syringe pump (rate 0.03 mL/h) into a previously degassed solution of xanthate (1 mmol) and olefin (2-3 equiv) in the solvent (1 mL or, in the case of less soluble compounds, in the minimum of solvent needed to obtain a clear solution), placed in a round bottom flask equipped with a stopper connected to a rubber balloon filled with argon. Air was introduced at the same time with a second syringe pump (10 mL/h). The reaction was monitored by TLC. Evaporation of the solvents under reduced pressure gave a residue that was purified by silica gel column chromatography.

O-Ethyl, *S*-{6-[(4-methylphenyl)sulfonyl]-4-oxo-1-[(trimethylsilyl)methyl] hexyl} dithiocarbonate [**19a**].

Xanthate **1a** (0.172 g, 0.496 mmol), allyltrimethylsilane **9** (0.142 g, 1.24 mmol, 2.5 equiv) in CH_2Cl_2 (1.3 mL). After 8 h (0.25 mL, 0.5 equiv of Et_3B was used), the reaction was worked-up as usually. Purification by flash chromatography gave compound **19a** as a colourless oil (0.162 g, 71% yield) without any traces of reduced product. ^{1}H NMR (300 MHz, $CDCl_3$): δ=7.76 (dd, ^3J=1.9 Hz, ^3J=8.2 Hz, 2H), 7.35 (d, ^3J =7.0 Hz, 2H), 4.60 (q, ^3J=7.1 Hz, 2H), 3.81 (m, 1H), 3.35 (t, ^3J=7.7 Hz, 2H), 2.84 (t, ^3J=7.1 Hz, 2H), 2.56 (q, ^3J=8.0 Hz, 2H), 2.43 (s, 3H), 2.07 (m, 1H), 1.76 (m, 1H), 1.40 (dt, ^3J=2.2 Hz, ^3J=7.1 Hz, 3H), 1.02 (2dd, ^3J=2.0 Hz, ^3J=7.0 Hz, 2H), 0.06 (s, 9H). ^{13}C NMR (75 MHz, $CDCl_3$): δ=214.3, 205.1, 144.8, 135.8, 129.9, 127.9, 69.7, 50.5, 48.1, 39.7, 35.2; 30.5, 23.5, 21.6, 13.7, -0.9. IR (neat): ν=1716, 1315, 1245, 1209, 1145, 1110, 1086, 1045, 838, 814. HRMS (ESI) [M+Na]+ Calcd. for $C_{20}H_{32}O_4S_3SiNa$: 483.1130. Found: 483.1085.
