# Supplementary material for: Part 3. Triethylborane-air: a suitable initiator for intermolecular radical additions of S-2-oxoalkyl-thionocarbonates (S-xanthates) to olefins
Source: Beilstein J Org Chem. 2007 Dec 13;3:47. doi: 10.1186/1860-5397-3-47 (PMC2213663; doi:10.1186/1860-5397-3-47)
Supplement: File 2 — Part 1. Reduction of S-alkyl-thionocarbonates and related compounds in the presence of trialkylboranes/air. Detailed procedures for preparation of new compounds and their spectroscopic data. [file Beilstein_J_Org_Chem-03-47-s002.pdf]

Supporting Information Available

Part 3.Triethylborane: A Suitable Initiator for Intermolecular Radical Additions of *S*-2-Oxoalkyl-thionocarbonates (Xanthates) to Olefins

Jean Boivin,[a] and Van Tai Nguyen*[a,b]*

a) Institut de Chimie des Substances Naturelles, C.N.R.S.,

Gif-sur-Yvette 91198, France.

b) National Institute of Medicines Materials

3B, Quang Trung, Hanoi, Vietnam

jean.boivin@icsn.cnrs-gif.fr *and* htqt.imm@hn.vnn.vn

Table of Contents

**General Experimental Methods**	**Page S3**
**Reagents and Starting Materials**	**Page S3**
**Preparation of Starting Substrates**	**Page S3**
**General Procedure for the Radical Addition of Xanthate to Olefin Initiated by Et_3B/air**	**Page S4**
**References**	**Page S20**

General Experimental Methods

Solvents were used as commerciale available. Experiments that required an inert atmosphere were carried out under dry argon in a flame dried glass system. Column chromatography was carried out on silica gel (230-400 mesh) by gradual elution with mixtures of n-heptane and ethyl acetate. TLC was carried out on using F_{254} SiO_2 coated aluminum plates (0,2 μm, analytical). Visualization was accomplished with UV light (254 nm), and by staining (and charring) of the TLC plates with a solution of p-anisaldehyde in sulfuric acid/ethanol. ^{1}H and ^{13}C NMR spectra were recorded in deuteriochloroform on 300 MHz spectrometers. The chemical shifts are reported δ unit, parts per million (ppm) relative to deuterated solvents (^{1}H, δ 7.269 ppm; ^{13}C, δ 77.23 ppm) or Me_4Si. Splitting pattern of an apparent multiplet associated with an averaged coupling constant were designated as s (singlet), d (doublet), t (triplet), q (quartet), m (multiplet), dd (doublet of doublet), dt (doublet of triplet), dq (doublet of quartet) and br (broadened). Coupling constants (*J* value) were reported in Hz unit. IR spectra were recorded on a FT-IR as a neat. ESI and high-resolution mass spectra (HRMS) were obtained with a LCT spectrometer. Previously reported reaction products were identified by spectral comparison.

Reagents and Starting Materials:

Triethylborane (1M solution in hexanes) was purchased from Aldrich Chemical Company, used without further purification, and stored under nitrogen atmosphere. All other commercially reagents were used as received.

Preparation of Starting Substrates:

Compound **7a** is a known compound:[1]

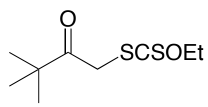

Compound **12** is a known compound:[2]

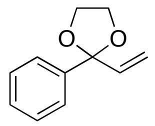

Compound **9** is a known compound:[3]

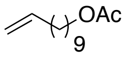

Compounds **1-6a** were prepared according to literature.[4, 5, 6]

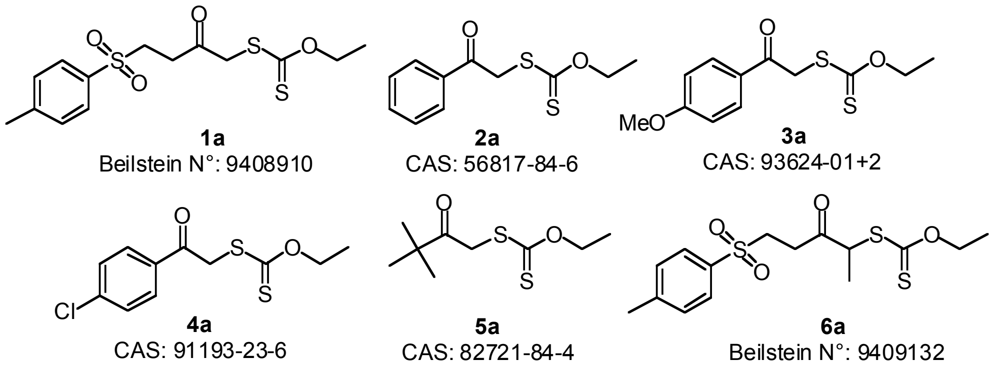

Compound **1b** is a known compound : [7]

General Procedure for the Radical Addition of Xanthate to Olefin Initiated by Et_3B/air:

Procedure A: reaction at room temperature

Triethylborane (1M solution in hexanes) was injected with the aid of a syringe pump (rate 0.03 mL/h) into a degassed solution of xanthate (1 mmol) and olefin (2-3 equiv) in the solvent (1 mL or, in the case of less soluble compounds, in the minimum of solvent needed to obtain a clear solution) placed in a round bottom flask equipped with a stopper connected to a rubber balloon filled with argon to equilibrate the pressures. Air was introduced into the solution at the same time (10 mL/h). The reaction was monitored by TLC. Evaporation of the solvent under reduced pressure afforded a residue that was purified by silica gel column chromatography.

Procedure B: reaction at "high" temperature

A solution of xanthate (1 mmol) and olefin (2-3 equiv) in the solvent (1 mL or, in the case of less soluble compounds, in the minimum of solvent needed to obtain a clear solution at reflux) was placed in a two-necked round bottom flask equipped with a condenser open to the air and heated to reflux. Triethylborane (1M solution in hexanes) was injected with the aid of a syringe pump (rate 0.03 mL/h) into the above solution. The reaction was monitored by TLC. Evaporation of the

solvent under reduced pressure afforded a residue that was purified by silica gel column chromatography.

***O*-Ethyl *S*-3-oxo-1-tosyltetradecan-6-yl carbonodithioate (1c)**

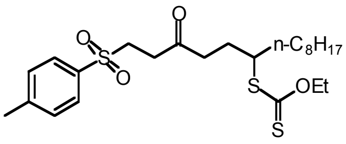

Chemical Formula: $C_{16}H_{21}O_4S_3$
Exact Mass: 373,06020
Molecular Weight: 373,53054

The title compound **1c** was prepared following the general procedure for the radical additions initiator by Et_3B at room temperature, starting from xanthate **1a** (0.189 g, 0.545 mmol) and 1-decene (0.197 g, 1.38 mmol, 2.5 equiv) in dichloromethane (1.5 mL). After 7 h (0.22 mL, 0.22 mmol, 0.4 equiv of Et_3B was used), the reaction was worked-up as usually. Flash chromatography gave compound **1c** as a colourless oil (0.169 g, 64% yield), and starting material **1a** (0.021 g, 11%) without any trace of reduced product **1b**. The spectral characteristics of compound **1c** were in agreement with the previously data.[7]

^{1}H NMR (300 MHz, $CDCl_3$): δ=0.85 (t, *J*=7 Hz, 3 H), 1.23 (m, 12 H), 1.40 (t, *J*=7 Hz, 3 H), 1.59 (m, 2 H), 1.75 (m, 1 H), 2.02 (m, 1 H), 2.44 (s, 3 H), 2.56 (t, *J*=7 Hz, 2 H), 2.85 (t, *J*=7.5 Hz, 2 H), 3.34 (t, *J*=7.5 Hz, 2 H), 3.66 (m, 1 H), 4.61 (q, *J*=7 Hz, 2 H), 7. 35 (d, *J*=8 Hz, 2 H), 7.76 (d, *J*=8 Hz, 2 H).

^{13}C NMR (75 MHz, $CDCl_3$): δ=214.43, 205.08, 129.86, 127.80, 69.81, 50.81, 50.40, 39.70, 35.11, 34.40, 31.64, 29.17, 28.99, 27.76, 26.58, 22.46, 21.46, 13.88, 13.58.

IR (film): ν =1050, 1087, 1112, 1150, 1215, 1317, 1453, 1719, 2855, 2926, 2954 cm^{-1}.

Preparation of 1-Hydroxy-5-[(4-methylphenyl)sulfonyl]-1-phenylpentan-3-one (1d)

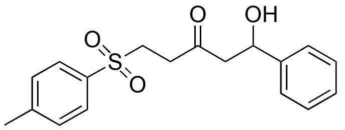

Chemical Formula: $C_{18}H_{20}O_4S$
Exact Mass: 332,1082
Molecular Weight: 332,4140

A solution of Et_3B (1.0 M in hexanes, 0.57 ml, 0.57 mmol, 1.2 equiv) was added to a solution of xanthate **1a** (0.165 g, 0.476 mmol) and benzaldehyde (0.071 g, 0.667 mmol, 1.4 equiv) in CH_2Cl_2 (2 mL) at room temperature under argon atmosphere. The mixture was stirred for 1 h then the rubber stopper was replaced by a drying tube filled with Drierite and $CaCl_2$. After 1 h, the solvent was concentrated in vacuo and the residue was purified by flash chromatography (heptane/EtOAc = 8/2 then 7/3) to furnish **1d** as a white solid (0.076 g, 48 %, m.p. 111.5-113°C), compound **1b** (0.012 g, 12%) and starting material **1a** (0.010 g, 6%).

^{1}H NMR (300 MHz, $CDCl_3$): δ=7.78 (d, *J*=8.3 Hz, 2 H), 7.40-7.25 (m, 7 H), 5.13 (dd, *J*=3.3 Hz, and *J*=9.3 Hz, 1 H), 3.38 (t, *J*=7.1 Hz, and *J*=7.7 Hz, 2 H), 2.93 (t, *J*=7.1 Hz, and *J*=7.7 Hz, 2 H), 2.88 (d, *J*=9.3 Hz, 1 H), 2.76 (dd, *J*=3.3 Hz, and *J*=9.3 Hz, 1 H), 2.47 (s, 3 H); ^{13}C NMR (75 MHz, $CDCl_3$): δ=205.9, 145.1, 142.6, 135.9, 130.0, 128.6, 128.0, 127.9, 125.6, 69.9, 51.5, 50.4, 36.1, 21.7; IR (neat): ν=3496, 1702, 1308, 1272, 1147, 1086, 1038, 1023, 748, 697, 685, and 647 cm^{-1}. HRMS (ESI): $[M+Na]^+$ Calcd. for $C_{18}H_{20}O_4SNa$: 345.0975. Found: 355.0947.

2-[(Ethoxycarbonothioyl)thio]-7-[(4-methylphenyl)sulfonyl]-5-oxoheptyl acetate (17a)

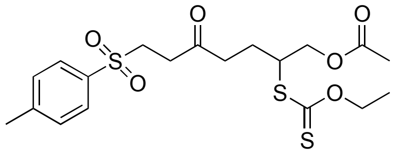

The title compound **17a** was prepared following the general procedure for the radical additions initiator by Et_3B at room temperature, starting from xanthate **1a** (0.185 g, 0.534 mmol) and allyl acetate (0.134 g, 1.34 mmol, 2.5 equiv) in dichloromethane (1.5 mL). After 9 h (0.27 mL, 0.27 mmol, 0.5 equiv of Et_3B was used), the reaction was worked-up as usually. Purification by chromatography gave compound **17a** as a colourless syrup (0.121 g, 51% yield), and starting material **1a** (0.038 g, 21%) without any trace of reduced product **17b**.

This compound was also prepared following the general procedure for the radical additions initiator by Et_3B at high temperature [$(CH_2Cl)_2$ reflux], starting from xanthate **1a** (0.346. g, 1.0 mmol) and allyl acetate (0.20 g, 2.0 mmol, 2 equiv) in 1,2-dichloroethane (1 mL). After 3.5 h (0.11 mL, 0.11 mmol, 0.11 equiv of Et_3B was used), the reaction was worked-up as usually. Purification by chromatography gave compound **17a** (0.347 g, 78% yield) without reduced product **17b.**

^{1}H NMR (300 MHz, $CDCl_3$): δ=7.76 (d, ^3J=8.3 Hz, 2H), 7.36 (d, ^3J=8.5 Hz, 2H), 4.63 (q, ^3J=7.1 Hz, 2H), 4.27 (dd, ^3J=5 Hz and $^3J'$=11.4 Hz, 1H), 4.19 (dd, ^3J=6.1 Hz and ^3J=11.4 Hz, 1H), 3.92 (m, 1H), 3.35 (t, ^3J=7.5 Hz, 2H), 2.86 (t, ^3J=7.5 Hz, 2H), 2.63 (t, ^3J=7.5 Hz, 2H), 2.45 (s, 3H), 2.10 (m, 1H), 2.05 (s, 3H), 1.81 (m, 1H), 1.41 (t, ^3J=7.1 Hz, 2H); ^{13}C NMR (75 MHz, $CDCl_3$): δ=212.90, 204.78, 170.66, 145.04, 135.88, 130.05, 128.00, 70.54, 65.62, 50.56, 48.90, 39.71, 35.25, 24.39, 21.67, 20.80, 13.75; IR (film): ν =1737, 1715, 1379, 1362, 1313, 1216, 1144, 1109, 1085, 1040, 813, 762, 722 cm^{-1}; HRMS (ESI): $[M+Na]^+$ Calcd. for $C_{19}H_{26}O_6S_3Na$ 469.0789. Found 469.0789.

***O*-Ethyl *S*-[1-methyl-1-((1*R*)-4-{5-[(4-methylphenyl)sulfonyl]-3-oxopentyl}cyclohex-3-en-1-yl)ethyl] dithiocarbonate (18a)**

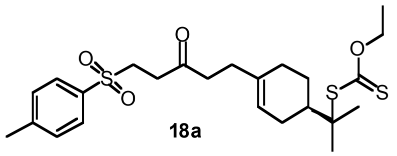

18a

Chemical Formula: $C_{24}H_{34}O_4S_3$
Exact Mass: 482,16192
Molecular Weight: 482,71936

The title compound **18a** was prepared following the general procedure for the radical additions initiator by Et_3B at room temperature, starting from xanthate **1a** (0.186 g, 0.537 mmol) and β-pinene (0.183 g, 1.34 mmol, 2.5 equiv) in dichloromethane (1.5 mL). After 9 h (0.27 mL, 0.27 mmol, 0.5 equiv of Et_3B was used), the reaction was worked-up as usually. Purification by flash chromatography gave compound **18a** as a yellow oil (0.114 g, 44%), the starting material **1a** (0.037 g, 20%), and product **18b** (0.023 g, 12%).

This compound was also prepared following the general procedure for the radical additions initiator by Et_3B at high temperature [$(CH_2Cl)_2$ reflux], starting from xanthate **1a** (0.405. g, 1.17 mmol) and β-pinene (0.318 g, 2.34 mmol, 2 equiv) in 1,2-dichloroethane (1.5 mL). After 6.5 h (0.2 mL, 0.2 mmol, 0.17 equiv of Et_3B was used), the reaction was worked-up as usually. Purification by flash chromatography gave compound **18a** (0.446 g, 79% yield), without any reduced product.

^{1}H NMR (300 MHz, $CDCl_3$): δ=7.75 (d, ^3J=8.2 Hz, 2H), 7.34 (d, ^3J=8.2 Hz, 2H), 5.30 (d, ^3J=4.0 Hz, 1H), 4.64 (q, ^3J=7.2 Hz, 2H), 3.33 (t, ^3J=7.5 Hz, 2H), 2.86 (t, ^3J=7.5 Hz, 2H), 2.50 (t, ^3J=7.5 Hz, 2H), 2.43 (s, 3H), 2.16 (t, ^3J=7.5 Hz, 2H), 2.10-1.76 (m, 7H), 1.44 (s, 6H), 1.41 (t, ^3J=7.2 Hz, 3H); ^{13}C NMR (75 MHz, $CDCl_3$): δ=214.17, 205.74, 144.89, 135.89, 135.71, 129.93, 127.88, 120.79, 69.28, 58.94, 50,52, 42.60, 41.00, 34.92, 30.79, 29.44, 26.92, 25.06, 24.71, 24.41, 21.58, 13.69; IR (neat): ν =1714, 1364, 1314, 1301, 1287, 1224, 1144, 1109, 1084, 1034, 813 cm^{-1}; HRMS (ESI): $[M+Na]^+$ Calcd. for $C_{24}H_{34}O_4S_3Na$: 505.1517. Found: 505.1509.

***O*-Ethyl *S*-{6-[(4-methylphenyl)sulfonyl]-4-oxo-1-[(trimethylsilyl)methyl]hexyl} dithiocarbonate (19a)**

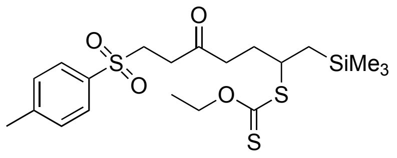

Chemical Formula: $C_{20}H_{32}O_4S_3Si$
Exact Mass: 460,1232
Molecular Weight: 460,7462

The title compound **19a** was prepared following the general procedure for the radical additions initiated by Et_3B/O_2 at room temperature, see Experimental Section in the Note.

The title compound **19a** was also prepared following the general procedure for the radical additions initiated by Et_3B/O_2 at high temperature (refluxing CH_2Cl_2), starting from xanthate **1a** (0.172 g, 0.496 mmol) and allyltrimethylsilane **9** (0.142 g, 1.24 mmol, 2.5 equiv) in CH_2Cl_2 (1 mL). After 4 h (0.15 mL, 0.15 mmol 0.3 equiv of Et_3B was used), the reaction was worked-up as usually. Purification by flash chromatography gave compound **19a** as a colourless oil (0.176 g, 77% yield) without any traces of reduced product.

^{1}H NMR (300 MHz, $CDCl_3$): δ=7.76 (dd, J=1.9 Hz, J=8.2 Hz, 2H), 7.35 (d, J=7.0 Hz, 2H), 4.60 (q, J=7.1 Hz, 2H), 3.81 (m, 1H), 3.35 (t, J=7.7 Hz, 2H), 2.84 (t, J=7.1 Hz, 2H), 2.56 (q, J=8.0 Hz, 2H), 2.43 (s, 3H), 2.07 (m, 1H), 1.76 (m, 1H), 1.40 (dt, J=2.2 Hz, J=7.1 Hz, 3H), 1.02 (2dd, J=2.0 Hz, J=7.0 Hz, 2H), 0.06 (s, 9H). ^{13}C NMR (75 MHz, $CDCl_3$): δ=214.3, 205.1, 144.8, 135.8, 129.9, 127.9, 69.7, 50.5, 48.1, 39.7, 35.2; 30.5, 23.5, 21.6, 13.7, -0.9. IR (neat): ν=1716, 1315, 1245, 1209, 1145, 1110, 1086, 1045, 838, 814. HRMS (ESI) $[M+Na]^+$ Calcd. for $C_{20}H_{32}O_4S_3SiNa$: 483.1130. Found: 483.1085.

***O*-Ethyl *S*-[6-[(4-methylphenyl)sulfonyl]-4-oxo-1-(trimethylsilyl)hexyl] dithiocarbonate (20a)**

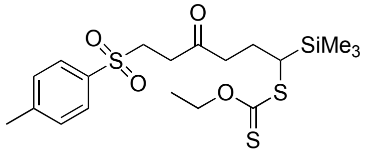

Chemical Formula: $C_{19}H_{30}O_4S_3Si$
Exact Mass: 446,1075
Molecular Weight: 446,7196

The title compound **20a** was prepared following the general procedure for the radical additions initiated by Et_3B/O_2 at room temperature, starting from xanthate **1a** (0.172 g, 0.496 mmol) and vinyltrimethylsilane **10** (0.124 g, 1.24 mmol, 2.5 equiv) in CH_2Cl_2 (1.3 mL). After 8 h (0.25 mL, 0.5 equiv of Et_3B was used), the reaction was worked-up as usually. Purification by flash chromatography gave compound **20a** as a colourless oil (0.127 g, 57% yield) without any traces of reduced product.

The title compound **20a** was also prepared following the general procedure for the radical additions initiated by Et_3B/O_2 at high temperature (refluxing CH_2Cl_2), starting from xanthate **1a** (0.172 g, 0.496 mmol) and vinyltrimethylsilane **10** (0.124 g, 1.24 mmol, 2.5 equiv) in CH_2Cl_2 (1 mL). After 1.5 h another portion of vinyltrimethylsilane **10** (0.124 g, 1.24 mmol, 2.5 equiv) was added. After 4 h (0.15 mL, 0.15 mmol, 0.3 equiv of Et_3B was used), the reaction was worked-up as usually. Purification by flash chromatography gave compound **20a** as a colourless oil (0.162 g, 73% yield) without any traces of reduced product.

^{1}H NMR (300 MHz, $CDCl_3$): δ=7.77 (d, J=8.2 Hz, 2H), δ=7.36 (d, J=8.4 Hz, 2H), 4.63 (q, J=7.1 Hz, 2H), 3.35 (dt, J=2.0 Hz, J=7.4 Hz, 2H), 3.09 (dd, J=3.4 Hz, J=11.2 Hz, 2H), 2.83 (dt, J=2.8

Hz, J=7.3 Hz, 2H), 2.59 (m, 2H), 2.45 (s, 3H), 2.14 (m, 1H), 1.61 (m, 1H), 1.42 (t, J=7.1 Hz, 3H), 0.09 (s, 9H). ^{13}C NMR (75 MHz, $CDCl_3$): δ=216.9, 205.6, 144.8, 135.9, 129.9, 127.9, 70.6, 50.5, 40.8, 36.3, 35.3, 24.3, 21.6, 13.8, -2.8. IR (neat): ν=1715, 1210, 1145, 1109, 1085, 1042, 837. HRMS (ESI) $[M+Na]^+$ Calcd. for $C_{19}H_{30}O_4S_3SiNa$: 469.0973. Found: 469.0993.

***O*-Ethyl *S*-{6-[(4-methylphenyl)sulfonyl]-4-oxo-1-[(4,4,5,5-tetramethyl-1,3,2-dioxaborolan-2-yl)methyl]hexyl} dithiocarbonate (21a)**

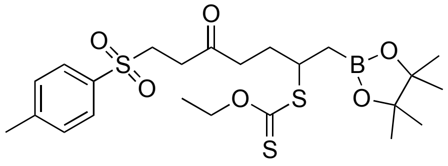

Chemical Formula: $C_{23}H_{35}BO_6S_3$
Exact Mass: 514,1689
Molecular Weight: 514,5264

The title compound **21a** was prepared following the general procedure for the radical additions initiated by Et_3B/O_2 at room temperature, starting from xanthate **1a** (0.172 g, 0.496 mmol) and allylboronic acid pinacol cyclic ester **11** (0.209 g, 1.24 mmol, 2.5 equiv) in CH_2Cl_2 (1.3 mL). After 8 h (0.25 mL, 0.5 equiv of Et_3B was used), the reaction was worked-up as usually. Purification by flash chromatography gave compound **21a** as a colourless oil (0.105 g, 41% yield) without any traces of reduced compound.

The title compound **21a** was also prepared following the general procedure for the radical additions initiated by Et_3B/O_2 at high temperature (refluxing CH_2Cl_2), starting from xanthate **1a** (0.172 g, 0.496 mmol) and allylboronic acid pinacol cyclic ester **11** (0.209 g, 1.24 mmol, 2.5 equiv) in CH_2Cl_2 (1 mL). After 4 h (0.15 mL, 0.15 mmol, 0.3 equiv of Et_3B was used), the reaction was worked-up as usually. Purification by flash chromatography gave compound **21a** as a colourless oil (0.138 g, 54% yield) without any traces of reduced product.

^1H NMR (300 MHz, $CDCl_3$): δ=7.76 (d, J=8.0 Hz, 2H), δ=7.36 (d, J=8.0 Hz, 2H), 4.62 (q, J=7.1 Hz, 2H), 3.88 (m, 1H), 3.35 (t, J=7.5 Hz, 2H), 2.56 (t, J=7.5 Hz, 2H), 2.44 (s, 3H), 2.03 (m, 1H), 1.90 (m, 1H), 1.40 (t, J=7.1 Hz, 3H), 1.22 (s, 12H). ^{13}C NMR (75 MHz, $CDCl_3$): δ=214.1, 205.1, 144.9, 135.9, 130.0, 127.9, 83.5, 69.7, 50.6, 47.1, 40.0, 35.2, 29.8, 24.72, 24.69, 21.6, 13.7. IR

(neat): ν=1716, 1365, 1315, 1212, 1140, 1109, 1086, 1045, 845, 814. HRMS (ESI) $[M+Na]^+$ Calcd. for $C_{23}H_{35}BO_6S_3Na$: 537, 1587. Found: 537,1572.

***S*-{1-(Diethoxymethyl)-6-[(4-methylphenyl)sulfonyl]-4-oxohexyl} *O*-ethyl dithiocarbonate (22a)**

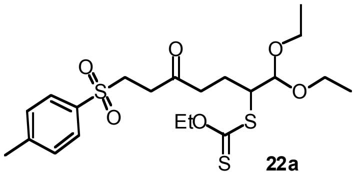

22a

Chemical Formula: $C_{21}H_{32}O_6S_3$
Exact Mass: 476,13610
Molecular Weight: 476,67018

The title compound **22a** was prepared following the general procedure for the radical additions initiator by Et_3B at high temperature (CH_2Cl_2 reflux), starting from xanthate **1a** (0.257 g, 0.742 mmol) and acrolein diethylacetal (0.34 mL, 0. 290 g, 2.23 mmol, 3.0 equiv) in dichloromethane (1.5 mL). After 9 h (0.27 mL, 0.27 mmol, 0.5 equiv of Et_3B was used), the reaction was worked-up as usually. Purification by flash chromatography gave compound **22a** as a pale yellow oil (0.263 g, 74%) and some starting material **1a** (0.015 g, 6%). The spectral characteristics of compound **22a** were in agreement with the previously reported data.[6]

^{1}H NMR (300 MHz, CDCl3): δ=7.69 (d, ^3J=8.1 Hz, 2H,), 7.30 (d, ^3J=8.1 Hz, 2H), 4.56 (q, ^3J=7.2 Hz, 2H), 4.46 (d, ^3J=2.9 Hz, 1H), 3.96 (m, 1H), 3.58 (m, 4H), 3.32 (dt, ^3J=1.3 Hz, ^3J=7.6 Hz, 2H), 2.82 (dt, ^3J=1.5 Hz, ^3J=7.6 Hz, 2H), 2.60 (dt, ^3J=1.8 Hz, ^3J=7.3 Hz, 2H), 2.43 (s, 3H), 2.19 (m, 1H), 1.81 (m, 1H), 1.39 (t, ^3J=7.2 Hz, 3H); 1.17 (m, 6H); ^{13}C NMR (65 MHz, $CDCl_3$): δ=214.0, 204.7, 144.3, 135.5, 129.5, 127.5, 103.4, 69.8, 64.0, 63.3, 52.8, 50.1, 39.3, 34.8, 21.7, 21.1, 14.7, 13.3; IR (film): ν =2977, 2929, 2896, 1718, 1597, 1444, 1406, 1316, 1219, 1148, 1111, 1053, 815 cm^{-1}.

***O*-Ethyl *S*-[4-oxo-4-phenyl-1-(2-phenyl-1,3-dioxolan-2-yl)butyl] dithiocarbonate (23a)**

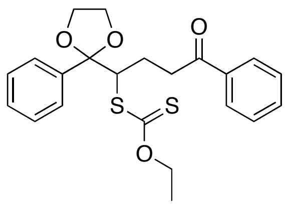

Chemical Formula: $C_{22}H_{24}O_4S_2$
Exact Mass: 416,1116
Molecular Weight: 416,5536

The title compound **23a** was prepared following the general procedure for the radical additions initiated by Et_3B at "high" temperature (CH_2Cl_2 reflux), starting from xanthate **2a** (0.182 g, 0.76 mmol) and phenyl vinyl dioxolane (0.267 g, 1.52 mmol, 2 equiv) in dichloromethane (1 mL). After 8 h (0.3 equiv of Et_3B was used), the reaction was worked-up as usually. Purification by flash chromatography gave compound **23a** as a colourless oil (0.161 g, 51% yield) and reduced product **2b** (0.011 g, 12%).

Compound 23a: ^{1}H NMR (300 MHz, $CDCl_3$): δ=7.9 (d, J=7.2 Hz, 2 H), 7.54 (m, 3 H), 7.46-7.31 (m, 5 H), 4.56 (m, 3 H), 4.12 (m, 2 H), 3.93-376 (m, 2 H), 3.12 (m, 2 H), 2.28 (m, 1 H), 1.91 (m, 1 H), 1.37 (t, J=7.1 Hz, 3 H); ^{13}C NMR (75 MHz, $CDCl_3$): δ=214.5, 199.3, 140.1, 136.8, 132.9, 128.5, 128.4, 128.1, 128.0, 126.4, 110.4, 70.2, 65.6, 65.2, 58.9, 35.8, 24.5, 13.7; IR (neat): ν=1682, 1447, 1209, 1042, 745, 689 cm^{-1}; MS (ESI): *m/z* = 439.0 $[M+Na]^+$. HRMS (ESI) $[M+Na]^+$ Calcd. for $C_{22}H_{24}O_4S_2Na$: 439.1014. Found: 439.0975.

***S*-[1-(Diethoxymethyl)-4-(4-methoxyphenyl)-4-oxobutyl] *O*-ethyl dithiocarbonate (24a)**

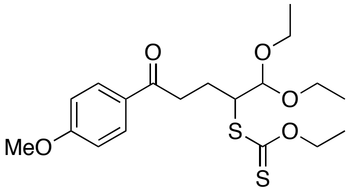

Chemical Formula: $C_{19}H_{28}O_5S_2$
Exact Mass: 400,1378
Molecular Weight: 400,5526

The title compound **24a** was prepared following the general procedure for the radical additions initiated by Et_3B at high temperature (refluxing CH_2Cl_2), starting from xanthate **3a** (0201. g, 0.743 mmol) and acrolein diethylacetal (0.290 g, 2.23 mmol, 3 equiv) in dichloromethane (1.5 mL). After 11 h (0.37 mL, 0.5 equiv of Et_3B was used), the reaction was worked-up as usually.

Purification by flash chromatography gave compound **24a** as a yellow oil (0.141 g, 47% yield) and compound **3b** (0.029 g, 26%).

This compound was also prepared following the general procedure for the radical additions initiator by Et_3B at high temperature [refluxing $(CH_2Cl)_2$], starting from xanthate **3a** (0270. g, 1.0 mmol) and acrolein diethyl acetal **13** (0.260 g, 2.0 mmol, 2 equiv) in 1,2-dichloroethane (1 mL). After 4 h (0.14 mL, 0.14 equiv of Et_3B was used), the reaction was worked-up as usually. Purification by flash chromatography gave compound **24a** as a yellow oil (0.239 g, 60% yield) without reduced product **3b**.

^{1}H NMR (300 MHz, $CDCl_3$): δ=7.94 (ddd, J=9 Hz, J=2.9 Hz, J=2.1 Hz, 2 H), 6.92 (ddd, J=9 Hz, J=2.9 Hz, J= 2.1 Hz, 2 H), 4.60 (m, 3 H), 4.15 (m, 1 H), 3.86 (s, 3 H), 3.82-3.50 (m, 4 H), 3.14 (m, 2 H), 2.42 (m, 1 H), 2.02 (m, 1 H), 1.38 (t, J=7.1 Hz, 3 H), 1.22 (dt, J=3.4 Hz, J=7.0 Hz, 6 H); ^{13}C NMR (75 MHz, $CDCl_3$): δ=214.8, 198.1, 163.3, 130.3, 130.0, 113.6, 104.0, 70.1, 64.4, 63.7, 55.4, 53.5, 35.4, 23.2, 15.2, 15.2, 13.7; IR (neat): ν=1673, 1597, 1256, 1214, 1168, 1110, 1045, 985, 828 cm^{-1}; MS (ESI): *m/z* = 423.1 $[M+Na]^+$. HRMS (ESI) $[M+Na]^+$ Calcd. for $C_{19}H_{28}O_5S_2Na$: 423.1276. Found: 423.1292.

10-[(Ethoxycarbonothioyl)thio]-15-[(4-methylphenyl)sulfonyl]-13-oxopentadecyl acetate (25a)

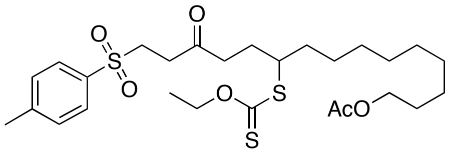

Chemical Formula: $C_{27}H_{42}O_6S_3$
Exact Mass: 558,2144
Molecular Weight: 558,8138

15-[(4-Methylphenyl)sulfonyl]-13-oxopentadecyl acetate (25b)

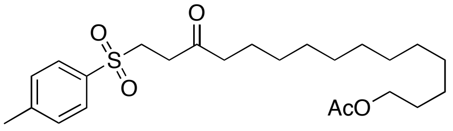

Chemical Formula: $C_{24}H_{38}O_5S$
Exact Mass: 438,2440
Molecular Weight: 438,6205

The title compound **25a** was prepared following the general procedure for the radical additions initiated by Et_3B/O_2 at high temperature (refluxing CH_2Cl_2), starting from xanthate **1a** (0.176 g, 0.51 mmol) and olefin **14** (0.216 g, 1.02 mmol, 2 equiv) in dichloromethane (1 mL). After 8 h (0.254 mL, 0.5 equiv of Et_3B was used), the reaction was worked-up as usually. Purification by flash chromatography gave compound **25a** as a yellow oil (0.177 g, 62% yield) and compound **25b** (0.044 g, 20%).

Compound 25a: ^{1}H NMR (300 MHz, $CDCl_3$): δ=7.70 (d, J=8.2 Hz, 2 H), 7.29 (d, J=8.5 Hz, 2 H), 4.56 (d, J=7.1 Hz, 2 H), 3.97 (t, J=6.8 Hz, 2 H), 3.62 (m, 1 H), 3.29 (t, J=7.4 Hz, 2 H), 2.79 (t, J=7.9 Hz, 2 H), 2.52 (brddd, 2 H), 2.38 (s, 3 H), 1.97 (s and m, 4 H), 1.77-1.49 (m, 5 H), 1.35 (t, J=7.1 Hz, 3 H), 1.20 (brs, 12 H); ^{13}C NMR (75 MHz, $CDCl_3$): δ=214.6, 205.2, 171.2, 144.9, 136.0, 130.0, 128.0, 70.0, 64.6, 51.0, 50.6, 39.9, 35.3, 34.6, 29.3, 29.2, 28.6, 28.0, 26.8, 25.9, 21.6, 21.0, 13.8; IR (neat): ν=2925, 1731, 1211, 1145, 1044 cm^{-1}; MS (ESI): *m/z*=281.2 $[M+Na]^+$. HRMS (ESI) [M+Na]+ Calcd. for $C_{27}H_{42}O_6S_3Na$: 581.2041 Found: 581.2060.

Compound 25b: ^{1}H NMR (300 MHz, $CDCl_3$): δ=7.71 (d, J=8.3 Hz, 2 H), 7.29 (d,J=8.3 Hz, 2 H), 3.98 (t, J=6.8 Hz, 2 H), 3.28 (t, J=7.5 Hz, 2 H), 2.81 (t, J=7.5 Hz, 2 H), 2.38 (s, 3 H), 2.34 (t, J=7.4 Hz, 2 H), 1.97 (s, 3 H), 1.5 (m, 4 H), 1.18 (s, 16 H); ^{13}C NMR (75 MHz, $CDCl_3$): δ=206.3, 171.2, 144.9, 136.1, 130.0, 128.0, 64.6, 50.6, 42.9, 35.0, 29.3, 29.2, 19.1, 28.6, 25.9, 23.7, 21.6, 21.0; IR (neat): ν=2913, 2847, 1723, 1706, 1253, 1241, 1142, 1084, 714, 767, 720, 686, and 639 cm^{-1}; MS (ESI): *m/z* = 461.2 $[M+Na]^+$. HRMS (ESI) $[M+Na]^+$ Calcd. for $C_{24}H_{38}O_5SNa$: 461.2338. Found: 461.2313.

***O*-Ethyl *S*-[6-[(4-methylphenyl)sulfonyl]-4-oxo-1-(2-phenyl-1,3-dioxolan-2-yl)hexyl] dithiocarbonate (26a)**

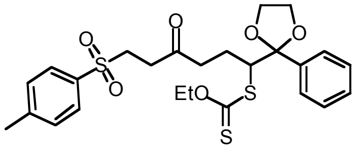

Chemical Formula: $C_{25}H_{30}O_6S_3$
Exact Mass: 522,1205
Molecular Weight: 522,6971

The title compound **26a** was prepared following the general procedure for the radical additions initiator by Et_3B at high temperature (CH_2Cl_2 reflux), starting from xanthate **1a** (0.260 g, 0.750 mmol) and phenyl vinyl dioxolane (0.264 g, 1.5 mmol, 2 equiv) in dichloromethane (1.5 mL).

After 12.5 h (0.37 mL, 0.37 mmol, 0.5 equiv Et_3B was used), the reaction was worked-up as usually. Purification by flash chromatography gave compound **26a** as a white solid (0.259 g, 66% yield). The spectral characteristics of **26a** prepared by this method were in agreement with the previously reported data.[6]

m.p. 100.6 – 101.6°C; ^{1}H NMR (300 MHz, $CDCl_3$): δ=7.75 (d, ^3J=8.2 Hz, 2H), 7.48 (d, ^3J=7.8 Hz, 2H), 7.35 (m, 5H), 4.59 (dq, ^3J=1.0 Hz, ^3J=7.1 Hz, 2H), 4.38 (dd, ^3J=3.7 Hz, ^3J=10.3, 1H), 4.15-3.97 (m, 2H), 3.88-3.71 (m, 2H) 3.29 (t, ^3J=7.2 Hz, 2H), 2.79 (m, 2H), 2.55 (m, 2H), 2.45 (s, 3H), 2.05 (m, 1H), 1.69 (m, 1H), 1.40 (t, ^3J=7.1 Hz, 3H); ^{13}C NMR (75 MHz, $CDCl_3$): δ=205.14, 144.92, 139.95, 135.94, 130.00, 128.50, 128.15, 128.00, 126.32, 110.27, 70.45, 65.55, 65.22, 58.85, 50.59, 39.88, 35.20, 23.76, 21.65, 13.78; IR (Nujol): ν =1048; 1111; 1144; 1223; 1298; 1376; 1461; 1721; 2854; 2924; 2954 cm^{-1}; MS (ESI): *m/z* (%): 545.1 (100) $[M+Na]^+$, 561.1 (20) $[M+K]^+$.

Diethyl {2-[(ethoxycarbonothioyl)thio]-7-[(4-methylphenyl)sulfonyl]-5-oxoheptyl} phosphonate (27a)

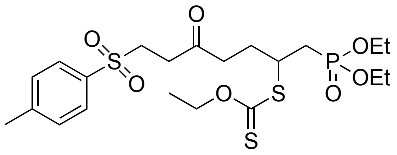

Chemical Formula: $C_{21}H_{33}O_7PS_3$
Exact Mass: 524,1126
Molecular Weight: 524,6513

The title compound **27a** was prepared following the general procedure for the radical additions initiated by Et_3B/O_2 at high temperature (refluxing CH_2Cl_2), starting from xanthate **1a** (0.17 g, 0.491 mmol) and diethyl allylphosphonate **15** (0.219 g, 1.23 mmol, 2.5 equiv) in CH_2Cl_2 (1 mL). After 4 h (0.15 mL, 0.15 mmol 0.3 equiv of Et_3B was used), the reaction was worked-up as usually. Purification by flash chromatography gave compound **27a** as a colourless oil (0.152 g, 59% yield) without any traces of reduced product.

^{1}H NMR (300 MHz, $CDCl_3$): δ=7.74 (d, J=8.3 Hz, 2H), 7.34 (d, J=8.0 Hz, 2H), 4.61 (t, J=7.1 Hz, 2H), 4.09 (m, 4H), 3.95 (m, 1H), 3.30 (t, 2H), 2.83 (dt, J=1.8 Hz, J=7.5 Hz, 2H), 2.59 (dt, J=1.8 Hz, J=7.5 Hz, 2H), 2.43 (s, 3H), 2.31 (m, 2H), 2.08 (m, 1H), 1.89 (m, 1H), 1.40 (t, J=7.1 Hz, 3H),

1.31 (t, J=7.1 Hz, 3H), 1.30 (t, J=7.1 Hz, 3H). ^{13}C NMR (75 MHz, $CDCl_3$): δ=212.6, 204.5, 144.9, 135.8, 129.9, 127.9, 70.1, 62.0, 61.9, 61.8, 50.5, 45.0, 39.7, 35.2, 32.5, 30.7, 27.4, 27.3, 21.6, 16.37, 16.31, 13.7. IR (neat): ν=1715, 1216, 1144, 1111, 1086, 1040, 1018, 959, 812 cm^{-1}. HRMS (ESI) $[M+Na]^+$ Calcd. for $C_{21}H_{33}O_7PS_3Na$: 547.1024. Found: 547.1058.

2-[(Ethoxycarbonothioyl)thio]-6,6-dimethyl-5-oxoheptyl acetate (28a)

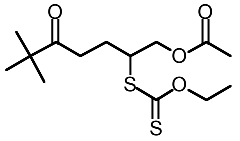

Chemical Formula: $C_{14}H_{24}O_4S_2$
Exact Mass: 320,1116
Molecular Weight: 320,468

The title compound **28a** was prepared following the general procedure for the radical additions initiator by Et_3B at high temperature (DCE reflux), starting from xanthate **5a** (0.206 g, 0.935 mmol, 1 equiv) and allyl acetate **7** (0.187 g, 1.87 mmol, 2 equiv) in 1,2-dichloroethane (1 mL). After 3.5 h (0.11 mL, 0.11 mmol, 0.11 equiv Et_3B was used), the reaction was worked-up as usually. Purification by flash chromatography gave compound **28a** as a yellow oil (0.23 g, 77% yield) without any reduced products. The spectral characteristics of **28a** prepared by this method were in agreement with the previously reported data.[8]

^{1}H NMR (300 MHz, $CDCl_3$): δ=7.52 (dd, ^3J=1.5 Hz and $^3J'$=7.8 Hz, 2H), 7.35 (3H, m), 4.59 (2H, m), 4.43 (dd, ^3J=3.7 Hz and $^3J'$=10.4 Hz, 1H), 4.09 (m, 2H), 3.82 (m, 2H), 2.60 (m, 2H), 2.07 (m, 1H), 1.67 (m, 1H), 1.41 (t, ^3J=7.0 Hz, 3H), 1.07 (s, 9H); ^{13}C NMR (75 MHz, $CDCl_3$): δ=215.00, 214.64, 140.16, 128.37, 128.05, 126.40, 110.40, 70.114, 65.54, 65.21, 58.76, 44.00, 33.77, 26.48, 24.00, 13.75; IR (neat): ν =2964, 1702, 1476, 1446, 1364, 1208, 1109, 1043, 774, 698, 640 cm^{-1}; HRMS (ESI): $[M+Na]^+$ Calcd. for $C_{20}H_{28}O_4S_2Na$: 419.1327. Found: 419.1332.

***S*-1-(Benzyloxy)-4-methyl-5-oxo-7-tosylheptan-2-yl *O*-ethyl carbonodithioate (29a)**

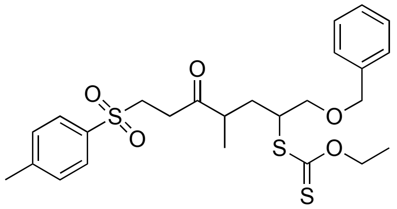

The title compound **29a** was prepared following the general procedure for the radical additions initiated by Et_3B at high temperature (DCE reflux), starting from xanthate **6a** (0.36 g, 1.0 mmol, 1 equiv) and allyl benzyl ether **16** (0.296 g, 2.0 mmol, 2 equiv) in 1,2-dichloroethane (1 mL). After 14 h (0.4 mL, 0.4 mmol, 0.4 equiv Et_3B was used), the reaction was worked-up as usually. Purification by flash chromatography gave compound **29a** as a colourless syrup (0.272 g, 54% yield) without any traces of reduced compound.

^{1}H NMR (300 MHz, $CDCl_3$): δ=7.78 (2d, 2H), 7.33 (m, 7H), 4.64 and 4.61 (2q, ^3J=7.1 Hz, 2H), 4.53 and 4.52 (2s, 2H), 3.94 and 3.88 (m, 1H), 3.68 and 3.56 (2m, 2H), 3.35 (m, 2H), 3.08-2.69 (m, 2H), 2.45 (s, 3H), 2.32 (m, 1H), 2.02 and 1.86 (2m, 1H), 1.60 (m, 1H), 1.42 and 1.40 (2t, ^3J=7.1 Hz, 3H), 1.12 and 1.11 (2d, ^3J=7.0 Hz, 3H); ^{13}C NMR (75 MHz, $CDCl_3$): 214.18, δ=208.97, 136.03, 130.00, 128.42, 127.98, 127.78, 127.75, 127.69, 127.65, 73.10, 73.01, 72.12, 72.04, 70.33, 50.65, 49.33, 48.67, 44.01, 43.77, 33.97, 33.92, 33.52, 33.42, 21.64, 17.63, 16.70, 13.77; IR (neat): ν =1712, 1596, 1453, 1313, 1212, 1146, 1109, 1086, 1040, 999, 813, 734, 696 cm^{-1}; HRMS (ESI): $[M+Na]^+$ Calcd. for $C_{25}H_{32}O_5S_3Na$: 531.1310. Found: 531.1319.

2-[(Ethoxycarbonothioyl)thio]-5-(4-methoxyphenyl)-5-oxopentyl acetate (30a)

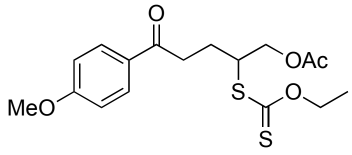

Chemical Formula: $C_{17}H_{22}O_5S_2$
Exact Mass: 370,0909
Molecular Weight: 370,4836

The title compound **30a** was prepared following the general procedure for the radical additions initiated by Et_3B/O_2 at high temperature (refluxing CH_2Cl_2), starting from xanthate **3a** (0.205 g, 0.758 mmol) and allyl acetate **7** (0.190 g, 0.19 mmol, 2.5 equiv) in dichloromethane (1 mL).

After 8 h (0.38 mL, 0.5 equiv of Et_3B was used), the reaction was worked-up as usually. Purification by flash chromatography gave compound **30a** as a yellow oil (0.137 g, 49% yield), and the compound **3b** (0.033 g, 29%). The spectral characteristics of **30a** prepared by this method were in agreement with the previously reported data.

This compound was also prepared following the general procedure for the radical additions initiated by Et_3B/O_2 at high temperature [refluxing $(CH_2Cl)_2$], starting from xanthate **4a** (0271. g, 1.0 mmol) and allyl acetate **8** (0.201 g, 2.0 mmol, 2 equiv) in 1,2-dichloroethane (1 mL). After 4 h (0.11 mL, 0.11 mmol, 0.11 equiv of Et_3B was used), the reaction was worked-up as usually. Purification by flash chromatography gave compound **22a** as a yellow oil (0.263 g, 71% yield) without any traces of compound **4b**.[9]

Compound 30a: ^{1}H NMR (300 MHz, $CDCl_3$): δ=7.95 (ddd, J=9.0 Hz, J=2.8 Hz, J=2/1 Hz, 2 H), 6.94 (ddd, J=9.0 Hz, J=2.8 Hz, J=2.1 Hz, 2 H), 4.63 (m, 2 H), 4.33 (m, 3 H), 4.01 (m, 1 H), 3.88 (s, 3 H), 3.13 (m, 2 H), 2.3 (m, 1 H), 2.08 (s, 3 H), 2.01 (m, 1 H), 1.40 (t, J=7.1 Hz, 3H); ^{13}C NMR (75 MHz, $CDCl_3$): δ= 212.8, 197.1, 170.5, 163.5, 130.2, 129.7, 113.7, 70.2, 65.6, 55.4, 49.0, 35.1, 25.1, 20.7, 13.6; IR (neat): ν=1738, 1673, 1598, 1212, 1168, 1110, 1041, 983, and 837 cm^{-1}; MS (ESI): *m/z* = 393.1 $[M+Na]^+$.

5-(4-Chlorophenyl)-2-[(ethoxycarbonothioyl)thio]-5-oxopentyl acetate (31a)

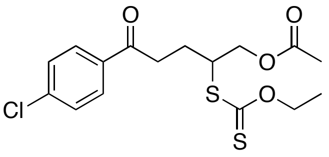

Chemical Formula: $C_{16}H_{19}ClO_4S_2$
Exact Mass: 374,0413
Molecular Weight: 374,9027

The title compound **31a** was prepared following the general procedure for the radical additions initiated by Et_3B/O_2 at high temperature [refluxing $(CH_2Cl)_2$], starting from xanthate **4a** (0.275 g, 1.0 mmol) and allyl acetate **7** (0.200 g, 2.0 mmol, 2 equiv) in 1,2-dichloroethane (1 mL). After 4 h (0.11mL, 0.11 mmol 0.11 equiv of Et_3B was used), the reaction was worked-up as usually. Purification by flash chromatography gave compound **31a** as a yellow oil (0.275 g, 73% yield) without any reduced products.

^{1}H NMR (300 MHz, $CDCl_3$): δ=7.88 (ddd, J=8.8 Hz, J=2.4 Hz, J=2.0 Hz, 2 H), 7.42 (ddd, J=8.8 Hz, J=2.4 Hz, J=2.0 Hz, 2 H), 4.62 (dq, J=1.4 Hz, J=7.1 Hz, 2 H), 4.36 (dd, J=11.4 Hz, J=4.9 Hz, 1 H), 4.27 (dd, J=11.4 Hz, J=6.2 Hz, 1 H), 4.06 (m, 1 H), 3.14 (dt, J=1.6 Hz, J=7.6 Hz, 2H), 2.31 (m, 1 H), 2.07 (s, 3 H), 2.01 (m, 1 H), 1.4 (t, 3 H); ^{13}C NMR (75 MHz, $CDCl_3$): δ=212.7, 197.4, 170.5, 139.6, 134.9, 129.4, 128.9, 70.3, 65.6, 48.9, 35.5, 24.9, 20.7, 13.6; IR (neat): ν=1738, 1682, 1588, 1211, 1090, 1041, 1012, 983 cm^{-1}; MS (ESI): *m/z* (%) = 397.0 $[C_{16}H_{19}Cl^{35}O_4S_2Na]^+$ (100), 399.0 $[C_{16}H_{19}Cl^{37}O_4S_2Na]^+$ (25). HRMS (ESI) $[M+Na]^+$ Calcd. for $C_{16}H_{19}Cl^{35}O_4S_2Na$: 397.0311. Found: 397.0301.

References

[1] Brown, M. D.; Gillon, D. W.; Meakins, G.; Whitham, G. H. *J.Chem. Soc. Perkin Trans.1* **1985**, 1623-1626.

[2] Gutzmann, J.; Messinger, P. *Arch. Pharm. (Weinheim Ger.)* **1995,** *328*, 6, 523-526

[3] Wang, P.; Blank, D. H.; Spencer, T. A. *J. Org. Chem.* **2004**, *69*, 2693-2702.

[4] Whitham, G. H.; Bridges, A. J. *J. Chem. Soc. Perkin. Trans. 1* **1975**, 1603-1606.

[5] Batanero, B.; Picazo, O.; Barba, F. *J. Org. Chem.* **2001**, *66*, 320-322. (b) Boivin, J.; Pothier, J.; Zard, S. Z., *Tetrahedron Lett.* **1999**, *40*, 3701-3704.

[6] Boivin J.; Jrad, R.; Juge, S.; Nguyen, V. T. *Org. Lett.* **2003**, *5*, 1645-1648.

[7] Pinnick, H.W.; Reynolds, M.A. *J. Org. Chem.* **1979,** *44*, 160-161.

[8] Part 1 Boivin, J.; Nguyen, V. T. *J. Org. Chem.* **2007**, submitted.

[9] Liard, A.; Quiclet-Sire, B.; Saicic, R. N.; Zard, S. Z. *Tetrahedron Lett.* **1997**, *38*, 1759-1762.
